# Consider Early ECT Treatment for Chronic Insomnia-Induced Suicidal Ideation

**DOI:** 10.1192/j.eurpsy.2023.2338

**Published:** 2023-07-19

**Authors:** A. D. Zhang, A. Pola, Y. Lin

**Affiliations:** 1 Virginia Tech Carilion School of Medicine; 2 Department of Psychiatry and Behavioral Science, Carilion Clinic, Roanoke, United States

## Abstract

**Introduction:**

Insomnia is a prevalent global health problem that affects 11.7% - 36% of the population (Grewal *et al.* Clinical Handbook of Insomnia Int 2017; 13 - 25). It is a risk factor for depression, poor quality of life, and accidents. Increasingly, insomnia has been identified as a suicide risk factor (Lin *et al.* BMC Psychiatry 2018: 18; 117) We present a case report of a 43-year-old male patient with insomnia-induced suicidal ideation (SI).

**Objectives:**

1. Learn the mechanism of insomnia-induced SI
2. Understand the current insomnia treatments
3. Discuss the possible mechanism of ECT treating insomnia-induced SI

**Methods:**

A 43-year-old single male with past psychiatric diagnoses of social anxiety, borderline personality disorder, chronic SI, and severe recurrent depression was admitted to inpatient due to intractable SI from insomnia. He failed trials on SSRIs/SNRIs, bupropion, trazodone, lithium, vortioxetine, quetiapine, zolpidem, and ketamine. The patient was initiated on electroconvulsive therapy (ECT) three times a week with 20mg vortioxetine and 100mg quetiapine for sleep initiation.

**Results:**

After 6-sessions, the patient’s mood, affect and sleep had improved considerably, and his suicidal ideations resolved. The patient was discharged with outpatient follow-up and ECT as rescue therapy. His sleep gradually improved to 4-6hrs/night and his mood was back at baseline.

**Conclusions:**

ECT is an effective treatment for refractory insomnia-induced SI, likely due to persistent REM suppression and reduced dendritic arborization and excitatory synapses in the amygdala (Doghramji *et al.* Sleep 2000; 23 - S16 - S20; Lahmeyer *et al.* Sleep Respiratory 1989; 18 346). Possible mechanisms for insomnia-induced SI include impaired decision-making, abnormalities in 5-HT function, or HPA dysfunction leading to a hyperarousal state and cortisol release (Chatzittofis *et al.* Euro Neuropsychopharmacology 2013; Elmenhorst *et al.* Sleep 2012; 35 1615 - 1623; Keilp *et al.* Psychol Medicine 2013; 43 539-551; Novati *et al.* 2008 Sleep; 31 1579 - 1585). Current insomnia treatments address underlying medical/psychological problems and non-pharmacologic and pharmacologic strategies. The predominant non-pharmacologic approach is Cognitive Behavioral Therapy Insomnia (CBT-I), such as relaxation techniques, sleep hygiene education, cognitive structuring, and sleep restriction (Rossman *et al.* American Journal of Lifestyle Medicine 2019; 13 544 - 547). Pharmacologic options include benzodiazepines, non-benzodiazepine hypnotics, tricyclic antidepressants, trazodone, and antihistamines (Saddichha *et al.* Annals of Indian Academy of Neurology 2010; 13 94-102). ECT should be strongly considered for the treatment of refractory insomnia-induced SI, and its early application may avoid rapid deterioration improving the quality of life.

**Disclosure of Interest:**

None Declared

